# Restoring gluconeogenesis by TEF inhibited proliferation and promoted apoptosis and immune surveillance in kidney renal clear cell carcinoma

**DOI:** 10.1186/s40170-023-00312-4

**Published:** 2023-08-08

**Authors:** Wenyuan Zhuang, Xiaokai Shi, Shenglin Gao, Xihu Qin

**Affiliations:** 1 Department of Urology, The Affiliated Changzhou Second People’s Hospital of Nanjing Medical University, Changzhou, 213003 China; 2 Gonghe County Hospital of Traditional Chinese Medicine, Hainan Prefecture, Qinghai Province, China; 3 Department of General Surgery, The Affiliated Changzhou Second People’s Hospital of Nanjing Medical University, Changzhou, 213003 China

**Keywords:** Kidney renal clear cell carcinoma, Gluconeogenesis, Proliferation, miR-4477b, Single-cell RNA sequencing

## Abstract

**Background:**

Kidney renal clear cell carcinoma (KIRC) is the major histological subtype of kidney tumor which covers approximately 80% of the cases. Although various therapies have been developed, the clinical outcome remains unsatisfactory. Metabolic dysregulation is a key feature of KIRC, which impacts progression and prognosis of the disease. Therefore, understanding of the metabolic changes in KIRC is of great significance in improving the treatment outcomes.

**Methods:**

The glycolysis/gluconeogenesis genes were analyzed in the KIRC transcriptome from the Cancer Genome Atlas (TCGA) by the different expression genes (DEGs) test and survival analysis. The gluconeogenesis-related miRNAs were identified by ImmuLncRNA. The expression levels of indicated genes and miRNAs were validated in KIRC tumor and adjunct tissues by QPCR. The effects of miR-4477b and PCK1 on cell proliferation and apoptosis were examined using the cell viability assay, cell apoptosis assay, and clone information. The interaction of miR-4477b with TEF was tested by the luciferase report gene assay. The different gluconeogenesis statuses of tumor cells and related signatures were investigated by single-cell RNA sequencing (scRNA-seq) analysis.

**Results:**

The 11 gluconeogenesis genes were found to be suppressed in KIRC (referring as PGNGs), and the less suppression of PGNGs indicated better survival outcomes. Among the 11 PGNGs, we validated four rate-limiting enzyme genes in clinical tumor patients. Moreover, restoring gluconeogenesis by overexpressing PCK1 or TEF through miR-4477b inhibition significantly inhibited tumor cell proliferation, colony formation, and induced cell apoptosis in vitro. Independent single-cell RNA sequencing (scRNA-seq) data analysis revealed that the tumor cells had high levels of PGNG expression (PGNG + tumor cells) represented a phenotype of early stage of neoplasia and prompted immune surveillance.

**Conclusions:**

Our study suggests that the deficiency of gluconeogenesis is a key metabolic feature of KIRC, and restoring gluconeogenesis could effectively inhibit the proliferation and progression of KIRC cells.

**Supplementary Information:**

The online version contains supplementary material available at 10.1186/s40170-023-00312-4.

## Background

Kidney renal clear cell carcinoma (KIRC) is the most common subtype of kidney tumor and accounts for about 80% of all cases, whose incidence has been increasing in recent years [1, 2]. KIRC is typically more aggressive and more likely to spread to other parts of the body than other types of kidney tumor [3]. The overall 5-year survival rate for patients with metastatic KIRC is low, at only 5–10% [3]. Thus, it is important for patients with KIRC to receive timely and appropriate treatment to increase the chances of a favorable outcome. The morphology of KIRC cells is characterized by the presence of high levels of lipids and glycogen, suggesting that altered metabolism of fatty acids and glucose may play a vital role in its development [4]. Recent researches have focused on the metabolic changes that occur in KIRC cells, including dysregulated glucose metabolism and increased glycolysis, which is known as the Warburg effect [4–7]. This metabolic change allows KIRC cells to generate more energy, support rapid cell growth, and contribute to the formation of an immune-suppression microenvironment. Thus, some researchers have suggested that kidney tumors, including KIRC, may be considered as a metabolic disease due to the involvement of various gene mutations in metabolic pathways, such as gluconeogenesis, glycolysis, the tricarboxylic acid (TCA) cycle, and glutamine metabolism [5, 8–10]. Gluconeogenesis, the reverse pathway of glycolysis, can antagonize aerobic glycolysis in cancer via the regulation of three key enzymes — phosphoenolpyruvate carboxykinase (PCK or PEPCK), fructose-1,6-bisphosphatase (Base), and glucose-6-phosphatase (G6Pase) [7]. And it is reported that overexpression of PCK1 promoted energy crisis and oxidative stress in liver cancer cells, leading to suppression of hepatocellular carcinoma [11, 12].

MicroRNAs (miRNAs) are small noncoding RNA molecules that play crucial roles in the regulation of gene expression [13]. Dysregulation of miRNAs has been implicated in the development and progression of various diseases, including cancer [13–15]. Some studies have identified miRNAs that are associated with altered metabolism in kidney tumor, including miR-199a-3p, miR-222, and miR-30c [16–18]. Other studies have suggested that targeting specific miRNAs may be a potential therapeutic approach for the treatment of kidney tumor [19].

However, the role of gluconeogenesis and how it is regulated by miRNA in kidney tumor is not yet fully understood. In this study, we employed the Cancer Genome Atlas (TCGA) KIRC transcriptome prolife to systematically investigate the role of glycolysis and gluconeogenesis pathways in KIRC. We identified an 11-gene gluconeogenesis signature, referring as PGNGs (protective gluconeogenesis genes), which was downregulated in tumor tissue, compared with normal ones. And the higher expression of the PGNGs stood for favorable prognosis in these patients. Remarkably, the PGNGs included 4 rate-limiting enzyme coding genes (G6PC, PCK1, PCK2, and FBP1) in gluconeogenesis pathway. Overexpression of PCK1 significantly inhibited the proliferation and survival in kidney tumor cell lines. In addition, we identified miR-4477b as an intrinsic inhibitor of KIRC gluconeogenesis by in silico analysis and further validated two kidney tumor cell lines. We found that FBP1 and TEF were direct targets of miR-4477b, while FBP1 served as the gluconeogenesis key enzymes and TEF transcriptionally regulated gluconeogenesis of PCK1 and PCK2. And the downregulation of miR-4477b significantly inhibited tumor cell clone formation and proliferation in vitro. An independent single-cell RNA sequencing (scRNA-seq) data revealed that the PGNGs + tumor cells represented a feature of early stage of neoplasia and high levels of immune cell cross talk. The findings of our study suggest that targeting the gluconeogenesis reprogramming, specifically by miR-4477b, showed clinical translational potential as a novel treatment for this disease.

## Methods

### KIRC transcriptome data acquisition and processing

The RNA sequencing data of TCGA KIRC was downloaded by R package TCGAbiolinks [20]. The R package biomaRt was employed to transform ENSEMBLE ID to gene symbol. Protein-coding and miRNA expression were obtained from split expression matrix whose ENSEMBLE ID was annotated as protein coding and miRNA, respectively. The related clinical information, including survival information, tumor stage, and so on, was downloaded from genomic data commons (GDC) data portal (https://gdc.cancer.gov/about-data/publications/pancanatlas).

### Bioinformatics analysis

Different expression genes (DEGs) between KIRC and control were determined by R package limma under the threshold of |log2FoldChange|> 1 and *p*-value < 0.05 [21]. Gene set enrichment analysis (GSEA) was accomplished by R package clusterProfiler function “GSEA” and visualized by function gseaplot2 of R package enrichplot [22]. The log2FoldChange was selected to order the genes. For single sample gene set enrichment analysis (ssGSEA), R package GSVA was applied to evaluate the overall expression level of gene set in single sample [23]. Survival analysis, including Cox hazard analysis and Kaplan–Meier (KM), was accomplished by R package survival. For KM, the median value was picked up for deciding high or low group. KM survival plot was visualized by “ggsurvplot” function of R package survminer. R package ImmuLncRNA was applied to identify the glycolysis-/gluconeogenesis-related miRNA [24]. Briefly, the partial correlation of selected miRNAs and all protein-coding genes was adjusted by tumor purity which was utilized as covariable. Then the ordered gene list based on the correlation coefficient was applied to GSEA pipeline to filter the vital miRNAs which were related to the focused gene list. The miRNAs whose lncRES score > 0.96 and *FDR* < 0.05 were considered significance. Transcription factor analysis was accomplished by R package RcisTarget [25]. The data of hg19-tss-centered-10 kb-7species.mc9nr.feather was picked up as gene-motif rankings database. MiRNA target gene was determined by R package miRNAtap which collected 5 online miRNA annotation databases. The target of miR-4477b was analyzed by TargetScan website (https://www.targetscan.org/vert_80/).

Protein–protein interaction (PPI) was identified by string database (https://string-db.org/) [26].

The KIRC scRNA-seq data was obtained from the published article (https://www.science.org/doi/10.1126/science.aat1699#supplementary-materials) [27]. The scRNA-seq data was applied the analysis pipeline of R package Seurat [28]. Briefly, the cell expression matrix was performed by quality control, normalization, scaled, dimensional reduction, and clustered. The cell type was automatically annotated by R package SingleR [29]. The cells from KIRC subjects were performed further analysis of trajectory and cell–cell interaction. The epithelial cells PGNGs status were determined by the expressed PGNGs genes number (> 3). The epithelial cells trajectory analysis was accomplished by R package CytoTRACE [30]. The cell–cell interaction was performed by R package CellChat [31].

### Clinical specimens preparation

This study was conducted in accordance with the Declaration of Helsinki and was approved by the Medical Ethics Committee of Changzhou Second People’s Hospital. Written informed consent was collected from all subjects. The 10 pairs of KIRC and adjacent normal tissue were obtained from Changzhou Second People’s Hospital and diagnosed by contrast-enhanced CT and pathological section. The specimens were immediately processed after collection.

### Cell culture and lentivirus transfection

Caki-1, 769-P, ACHN, and HEK-293 cell lines were obtained from National Collection of Authenticated Cell Cultures of China. And the cells were cultured in RPMI 1640 GlutaMAX-l medium or Dulbecco’s Modified Eagles Medium (DMEM) GlutaMAX-l medium and supplemented with 10% fetal calf serum (FCS), 1% sodium pyruvate, and 1% penicillin/streptomycin (Thermo, USA). All cells were cultured at 37 °C 5% CO2 in a humidified incubator. The lentivirus of PCK1 overexpression and control vector was provided by OBiO Biotechnology (China), and the miR-4477b mimic and inhibitor were synthesized by Thermo (USA). Generally, the cells were transfected with lentivirus and polybrene (Beyotime, China) with a MOI of 10. Forty-eight hours after transfection, the overexpression of target gene was confirmed by QPCR.

### QPCR

Total RNA of the tissue and cells was extracted using RNA Isolator Total RNA Extraction Reagent (Vazyme, China), and the RNA was reverse-transcribed by PrimeScript RT Master Mix (TaKaRa, Japan), according to the manufacturer’s instructions. The QPCR assay was tested by SYBR Green Mix (Life Technology, USA) and quantified by ABI 7500 Real-Time PCR System (Applied Biosystems, USA). The relative expression of each gene was normalized to Actin by the 2∆Ct method. The specific primer sequences are listed in Table 1.

**Table 1:** Specific primer sequences

Gene	Forward primer	Reverse primer	
ACTB	CATGTACGTTGCTATCCAGGC	CTCCTTAATGTCACGCACGAT	
ALDOB	TGTCTGGTGGCATGAGTGAAG	GGCCCGTCCATAAGAGAAACTT	
FBP1	GAACCGGAGAAAAGGGGTAAAT	GTTCCAACGGACACAAGGCA	
PFKM	GGTGCCCGTGTCTTCTTTGT	AAGCATCATCGAAACGCTCTC	
PCK1	AAAACGGCCTGAACCTCTCG	ACACAGCTCAGCGTTATTCTC	
G6PC1	CACTTCCGTGCCCCTGATAA	AGTATACACCTGCTGTGCCC	
TEF	GCGAGACGCGCCTTGATAA	ATCGTAGGGGATGGTCTTGTC	
PCK2	GGCTGAGAATACTGCCACACT	ACCGTCTTGCTCTCTACTCGT	
	Forward primer	Universal reverse primer	Stem-loop
MIR514B	GTTTTCTCAAGAGGGAGGC	GTGCAGGGTCCGAGGT	GTTGGCTCTGGTGCAGGGTCCGAGGTATTCGCACCAGAGCCAACATGATT
MIR4477B	GTTGGGATTAAGGACATTTGTG	GTGCAGGGTCCGAGGT	GTTGGCTCTGGTGCAGGGTCCGAGGTATTCGCACCAGAGCCAACATCAAT

### Luciferase report gene assay

HEK293 cells were seeded (1 × 10^4^/well) in 96-well plates 24 h before transfection. The wild-type (WT) or mutant (Mut) UTR of TEF 3′ UTR were inserted to the MCS of Promoterless NanoLuc Genetic Reporter Basic Vectors (Promega, USA). These plasmids were transfected with Lipofectamine 3000 into cells with miR-4774b mimic or scramble control. Forty-eight hours later, cells were lysed, and luciferase activity was measured using the Dual-Luciferase Reporter Assay System (Promega, USA).

### Cellular glucose and lactate assay

The cellular glucose and lactate assay kits were purchase from Abcam (CA, USA). The assays were performed according to the manufacturer’s instruction. Briefly, the cells were starved for 24 h in a low glucose (1 g/L) media. After wash with PBS twice on ice, the cell concentration was counted, and then the cells were lysed. The cellular content was then diluted 10-folds by PBS before applied to the assay. The level of OE or inhibitor samples was normalized to the control group.

### Cell viability assays

Cells transfected with different transfection were seeded at 3000 cells/well in a removable 96-well plates in triplicate. Then the plates were incubated at 37 °C. Every 12 h, the lanes of target cells were removed from the plate, and the cell viability was detected using Cell Counting Kit-8 (Vazyme, China). The optical absorbance of each well at 450 nm was measured using a microplate reader (Bio-Rad Laboratories, USA). The relative cell viability was normalized according to the control wells.

### Cell apoptosis assay

The level of apoptosis of the cells was analyzed by flow cytometry. Tumor cells were transfected with PCK1 OE lentivirus, miR-4477b inhibitor, or the corresponding NCs. After 48 h of incubation, cells were collected and stained with Annexin V/FITC kit (Beyotime, China). Finally, the cells were analyzed by a flow cytometry machine (BD, USA).

### Colony formation assay

For the colony formation assay, cells were transfected with indicated lentivirus or control. Then the cells were seeded in a 12-well plate (100 cells per well) and left to attach for 24 h and cultured for 10–12 days. At the end of the experiment, surviving colonies were fixed with 4% formaldehyde, washed with PBS trice, and then stained with crystal violet solution. The percentage of colonies relative to controls was calculated.

### Statistical analyses

R (Version 4.1.2) was used for all statistical tests. Spearman correlation analyses were conducted using R. The statistical analysis of ssGSEA scores between different conditions was performed using the Wilcoxon test. The significance of QPCR results was determined using a Student’s *t*-test, assuming a normal distribution. *P*-values of multiple tests were adjusted by false discovery rate (FDR). All *P*-values were considered significant if less than 0.05.

## Results

### Glycolysis/gluconeogenesis metabolic reprogram was essential for KIRC prognosis in TCGA

**Fig. 1:**
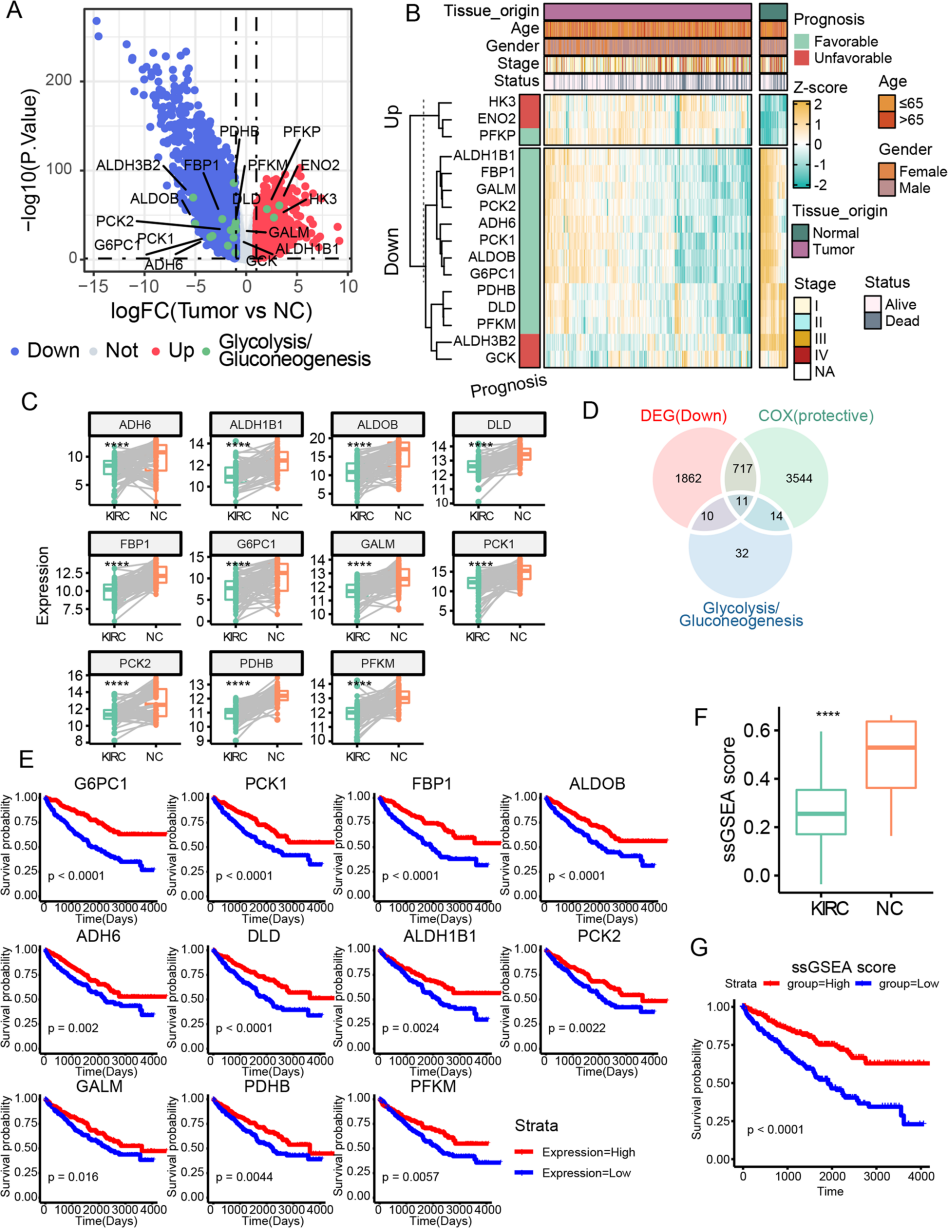
Reprogram of glycolysis and gluconeogenesis was crucial for KIRC prognosis. **A** Volcano plot of DEGs in the comparison of KIRC vs NC. The color indicated the upregulated (red), downregulated (blue), nonsignificant (gray), and glycolysis/gluconeogenesis (green) genes in KIRC. **B** Heatmap of indicated glycolysis/gluconeogenesis genes in TCGA KIRC mRNA profile. The prognosis of each gene was annotated on the left side by the univariate Cox hazard analysis. **C** Boxplot of PGNG genes from paired TCGA KIRC patients and adjacent normal tissues. **D** Venn diagram of downregulated DEGs, protective genes, and glycolysis/gluconeogenesis genes. **E** KM plot of PGNG genes. The expression group was determined by median value of each gene. **F** Boxplot of ssGSEA score of PGNG in KIRC and adjacent normal tissues. **G** KM plot of ssGSEA score of PGNG. The group was determined by median value of ssGSEA score. *P < 0.05, **P < 0.01, ***P < 0.001, ****P < 0.0001

We systematically analyzed the expression and survival significance of all 67 glycolysis/gluconeogenesis-related genes annotated from the Kyoto Encyclopedia of Genes and Genomes (KEGG) database in TCGA KIRC transcriptome dataset [32]. Of these genes, 28 were differentially expressed between tumor and adjacent normal tissues, with 21 being low expressed in the tumor (Fig. 1A). Interestingly, the rate-limiting enzyme-coding genes in the glycolysis pathway showed inconsistent expression pattern in tumor cells compared with normal: HK3 and PFKP were overexpressed, while PFKM was downregulated (Fig. 1A, B). It was worthy to note that PKM, another gene which limited glycolysis, was also slightly overexpressed in KIRC (fold change = 1.33). In contrast, the rate-limiting enzyme genes in the gluconeogenesis pathway (G6PC1, FBP1, PCK1, and PCK2) were consistently suppressed in KIRC (Fig. 1A, B). Moreover, the paired examination of abovementioned genes between KIRC tumor and paired adjacent tissue also confirmed these observation (Fig. 1C), indicating a drastic glycolysis/gluconeogenesis reprogram in KIRC.

Next, the univariate Cox hazard analysis demonstrated that 16 genes out of the 28 glycolysis/gluconeogenesis DEGs were prognostic significant, while 12 genes were protective, meaning their higher expression was associated with better survival outcomes (Fig. 1B, D and Fig. S1A). Interestingly, most of protective genes were downregulated in tumor tissue except for PFKP (Fig. 1B), suggesting that their deficiency might contribute to the initiation, development, and prognosis of KIRC. The survival significance of these genes was confirmed by KM analysis (Fig. 1E). We extracted these downregulated and protective genes (ADH6, ALDH1B1, ALDOB, DLD, FBP1, G6PC1, GALM, PCK1, PCK2, PDHB, PFKM) and named them protective gluconeogenesis genes (PGNGs). Consistently, the ssGSEA reflecting the overall levels of PGNGs signature was decreased in KIRC and had a better prognosis than individual genes (Fig. 1F, G and Fig. S1B). To better understand the biological connection among PGNGs, the protein–protein interaction (PPI) was constructed. In the network, these genes showed a close relationship with each other (Fig. S1C and D), and the 4 rate-limiting enzyme genes of gluconeogenesis pathway showed a comparable high degree (number of connections) in the PPI network.

### Promotion of gluconeogenesis inhibited renal carcinoma cells proliferation and clone formation and induced cell apoptosis

**Fig. 2:**
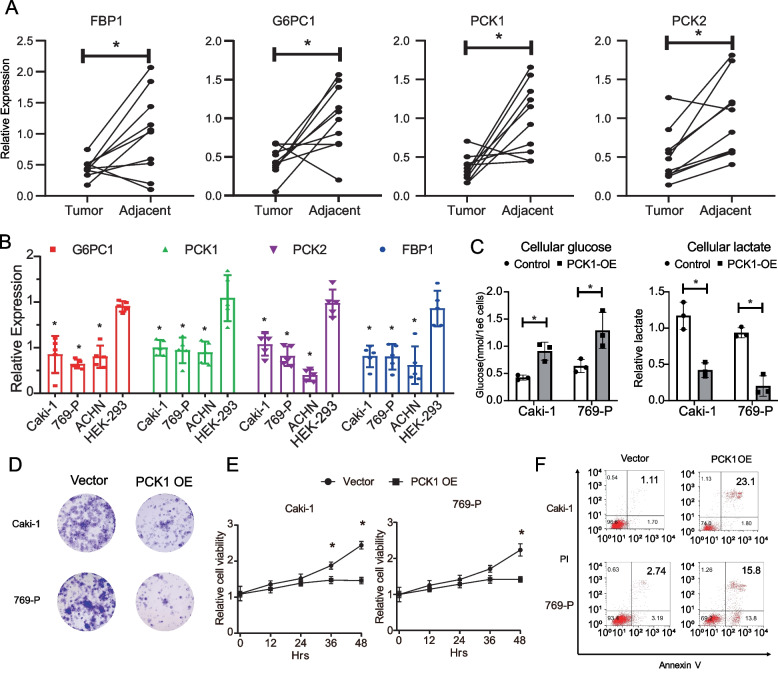
Promoting gluconeogenesis inhibited the proliferation, clone formation, and induced apoptosis in renal carcinoma cells. **A** The relative expression of indicated genes in tumor and adjacent tissues was tested by QPCR. *N* = 10. **B** The relative expression of indicated genes in kidney tumor and normal cell lines was tested by QPCR. *N* = 5. **C** The levels of cellular glucose (left) and lactate (right) in Caki-1 and 769-P cells. *N* = 3. **C** Colony formation assay comparing Caki-1 and 769-P transfected with PCK1 overexpression lentivirus control vector. **D** After transfected with PCK1 OE or control vector, the cell viability of Caki-1 and 769-P cells was tested at indicated time points by CCK8 assay. **E** The level of apoptosis of Caki-1 and 769-P cells transfected with PCK1 OE or control vector was analyzed by Annexin V and PI staining by flow cytometry. *P < 0.05

To confirm the aforementioned findings, we assessed the transcription levels of the rate-limiting enzyme genes of gluconeogenesis (G6PC1, FBP1, PCK1, and PCK2) in 10 paired KIRC tumor and adjacent normal tissues. We observed significant downregulation of all four genes in the tumor tissues compared to the para-cancerous normal tissues (Fig. 2A). Subsequently, we examined the role of the rate-limiting enzyme genes of gluconeogenesis at cellular level. In three renal carcinoma cell lines (Caki-1, 769-p, and ACHN) and one normal kidney cell line (HEK-293), we observed a significant decrease of gluconeogenesis genes in tumor cell lines at transcription level (Fig. 2B). To reverse the inhibited status of gluconeogenesis in cancer cells, we introduced the key enzyme PCK1 into two cancer cell lines using lentivirus. The overexpression of PCK1 led to an increase of cellular glucose levels and a decrease in lactate levels in both cancer cell lines (Fig. 2C). Additionally, this overexpression significantly inhibited cell growth and reduced colony formation in both tumor cell lines (Fig. 2D, E). Furthermore, the overexpression of PCK1 also induced a significant amount of cell apoptosis, as tested by flow cytometry (Fig. 2E). These results suggested that restoring gluconeogenesis could restrict the pro-survival capacity of cancer cells.

### Mir-4477b was predicted to be the suppressor of gluconeogenesis by targeting TEF and FBP1

**Fig. 3:**
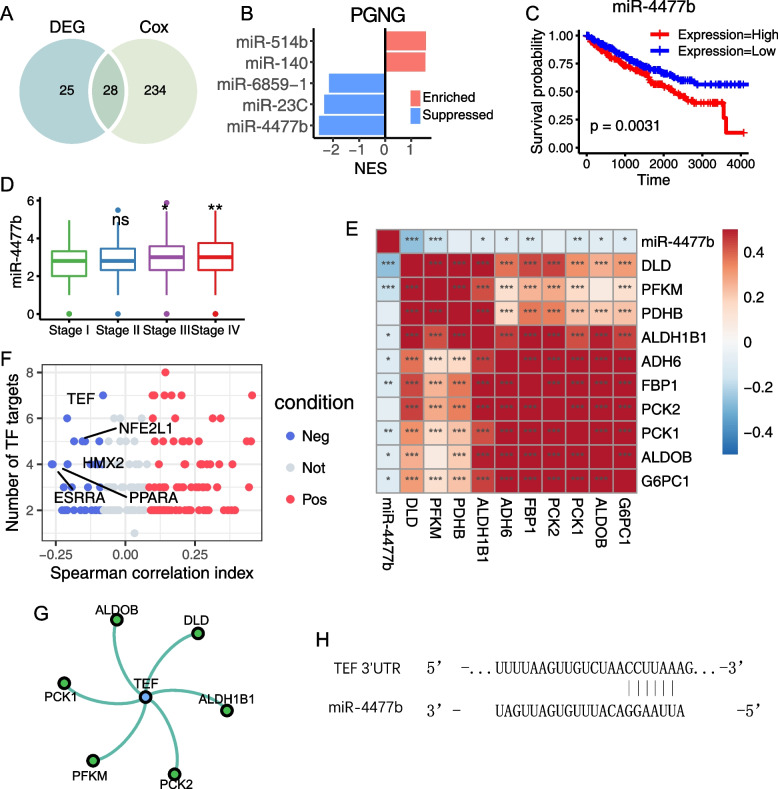
Mir-4477b was predicted to suppress gluconeogenesis by targeting TEF and FBP1. **A** Venn diagram of DE miRNAs and prognostic miRNAs. **B** Bar plot of ImmuLncRNA NES of miRNAs which targeted PGNG. The color indicated the regulation of each miRNA to PGNG, with the condition of enriched (red) or suppressed (blue). **C** KM plot of miR-4477b. The group was determined by median value of miR-4477b expression. **D** Boxplot of miR-4477b among different tumor stages. The stages II, III, and IV were compared to I, respectively. **E** Pearson correlation heatmap of miR-4477b and PGNG genes. **F** Dot plot of miR-4477b targeted TFs. The color indicated the correlation condition of TFs with miR-4477b. **G** Network of TEF and predicted targeted PGNG genes. **H** The predicted binding sites between miR-4477b and the 3′ UTR of TEF mRNA. *P < 0.05, **P < 0.01, ***P < 0.001

To investigate the contribution of miRNAs to the glucose metabolism reprogramming in KIRC, we analyzed miRNAs that suppressed PGNGs using ImmuLncRNA, which utilized the tumor purity-adjusted partial correlation between noncoding RNA and mRNA and subjected it to a GSEA-based pipeline. We identified 28 potential candidate miRNAs that were significantly altered both in DEG and survival analysis (Fig. 3A). Out of these, five miRNAs were significantly correlated with PGNGs, and three were predicted to inhibit PGNGs in KIRC, as shown in Fig. 3B. Notably, miR-4477b and miR-514b had the highest negative and positive normalized enrichment score (NES) for PGNGs, respectively, while miR-4477b had the highest absolute NES (Fig. 3B). This result implied that miR-4477b was the vital miRNA in restricting gluconeogenesis. In KIRC, patients with higher levels of miR-4477b showed poorer prognosis (Fig. 3C). Additionally, the expression of miR-4477b was higher in later tumor stages (TNM III and IV) compared to TNM I (Fig. 3D), suggesting its key role in cancer development.

To further elucidate the regulation mechanism of miR-4477b on PGNGs, we investigated the possible molecular interaction of miR-4477b with PGNGs. By utilizing five online miRNA databases, we identified FBP1 as the direct target of miR-4477b. Since the PGNGs exhibited a broadly positive correlation with each other (Fig. 3E), we postulated that PGNGs might be regulated by a common transcription factor (TF) which was targeted by miR-4477b. In Fig. 3F, we found that 21 out of 186 predicted transcription factors were negatively correlated with miR-4477b. Among them, TEF was relatively significant in both Spearman correlation coefficient (− 0.21) and target gene numbers (6). Interestingly, PCK1 and PCK2, two genes encoding rate-limiting enzymes in gluconeogenesis, were predicted to be transcriptionally regulated by TEF (Fig. 3G). Besides, the sequence alignment revealed a potential binding site between miR-4477b and the 3′ UTR of TEF mRNA (Fig. 3H). Thus, we hypothesized that TEF was the main target of miR-4477b to suppress gluconeogenesis in kidney tumor cells.

### Targeting miR-4477b inhibited the colony formation and survival in renal cells

**Fig. 4:**
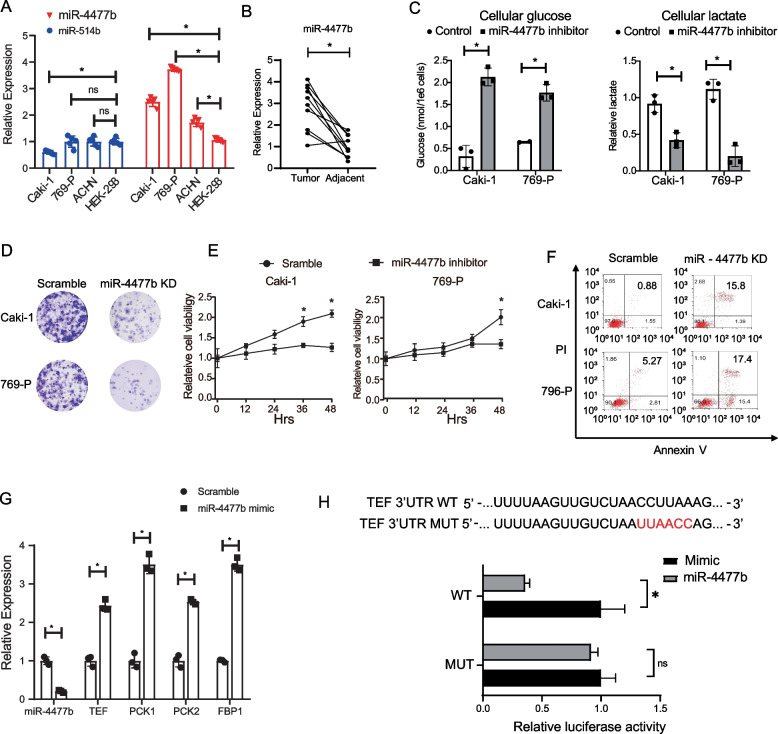
Targeting miR-4477b inhibited the proliferation, clone formation, and induced apoptosis in renal carcinoma cells. **A** The relative expression of miR-4477b and miR-514b was tested in tumor and normal kidney cell lines. *N* = 5. **B** The relative expression of miR-4477 was tested in tumor tissue and adjacent normal tissue in patient samples. *N* = 10. **C** The levels of cellular glucose (left) and lactate (right) in Caki-1 and 769-P cells. *N* = 3. **D** Colony formation assay was performed in Caki-1 and 769-P cells, transfected with miR-4477b antisense inhibitor or scramble inhibitor. **D** After transfected with miR-4477b antisense inhibitor or scramble inhibitor, the cell viability of Caki-1 and 769-P cells was tested at indicated time points by CCK8 assay. **E** The level of apoptosis of Caki-1 and 769-P cells transfected with miR-4477b antisense inhibitor or scramble inhibitor was analyzed by Annexin V and PI staining by flow cytometry. **F** The relative expression of indicated genes was test by QPCR in Caki-1 cells transfected with miR-4477b antisense inhibitor or scramble inhibitor. **G** Upper panel: the WT and mutation of the 3′-UTR region of TEF gene. The mutation of the binding site of miR-4477b was shown in red. Lower panel: Hek293 cells were transfected with the WT and MUT TEF 3′-UTR-Luc vector together with Renilla luciferase and miR-4477b mimic. Cells were harvested for luciferase assays 48 h after transfection. The relative luciferase activity was normalized by Renilla luciferase activity. *P < 0.05

To comprehensively investigate the regulatory role of miRNAs in gluconeogenesis and its underlying molecular mechanism, we analyzed the expression levels of miR-4477b in both cell lines and clinical patient tissues. As shown in Fig. 4A, miR-4477b was significantly upregulated in cancer cell lines, whereas miR-514b was not significantly altered between cancer and normal cell lines. Similarly, the level of miR-4477b was also upregulated in tumor tissues from clinical samples (Fig. 4B). To explore the functional role of miR-4477b in gluconeogenesis, we downregulated miR-4477b using a single-stranded oligo RNA inhibitor in both Caki-1 and 796-P cells. This led to the upregulation of cellular glucose levels and downregulation of lactate levels and decreased cell proliferation and colony formation (Fig. 4C–E). Furthermore, inhibition of miR-4477b mimicked the pro-apoptotic effect of PCK1 overexpression (Fig. 4F), suggesting its role in regulating gluconeogenesis. To validate the direct regulation effect of miR-4477b on gluconeogenesis genes, the overexpression of miR-4477b in HEK293 cells significantly downregulated the expression of TEF, FBP1, PCK1, and PCK2 (Fig. 4G). Next, we performed a dual-luciferase reporter assay too confirm the regulatory effect of miR-4477b on TEF. As shown in Fig. 4H, overexpression of miR-4477b significantly inhibited the luciferase activity of TEF in wild-type (WT) but not mutant (MUT) cells, indicating that TEF is directly targeted by miR-4477b.

### PGNG + epithelial cells were characterized by high levels of differential and chemotaxis in scRNA-seq analysis of KIRC tissue

**Fig. 5:**
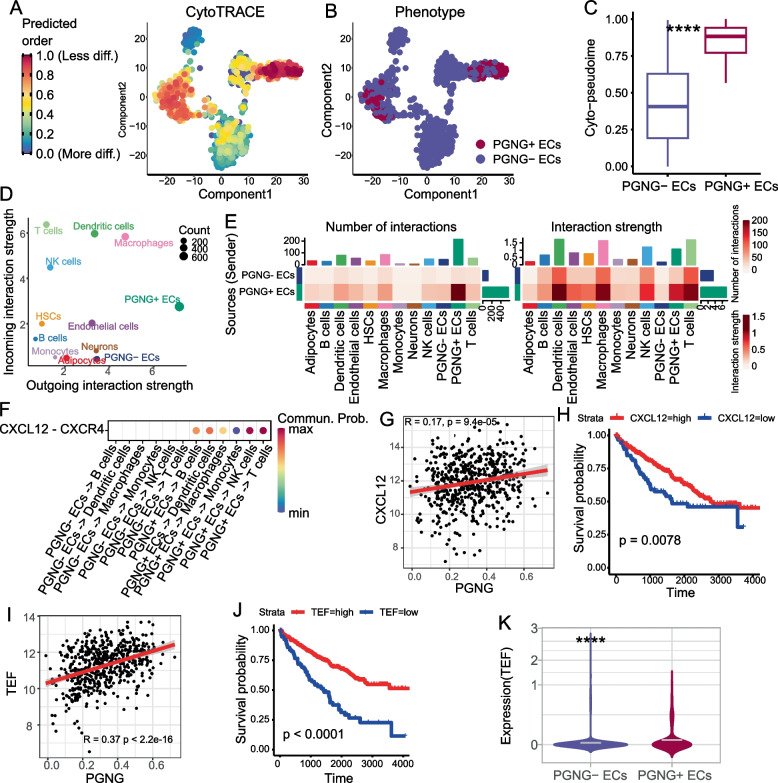
PGNG + ECs were well-differentiated and hyper-chemotic in bulk- and scRNA-seq. **A**–**B** T-SNE plots of epithelial cells of KIRC tumor scRNA-seq which were colored by CytoTRACE pseudotime (**A**) and PGNG expression status (**B**). **C** Boxplot of CytoTRACE pseudotime between PGNG statuses. **D** Dot plot of cells incoming and outgoing interaction strength calculated by CellChat. **E** The interaction number (left) and strength (right) from PGNG + / − ECs to all cells. **F** Dot plot of CXCL12-CXCR4 interaction from PGNG + / − ECs to immune cells in KIRC scRNA-seq dataset. The dot color represents the communication probability. **G** Dot plot of CXCL12 and PGNG ssGSEA score in TCGA KIRC transcriptome dataset. The Spearman correlation coefficient and *p*-value were annotated in the left-top corner. **H** KM plot of the CXCL12 gene. The expression group was determined by median value of CXCL12 gene. **I** Dot plot of TEF and PGNG ssGSEA score in TCGA KIRC transcriptome dataset. The Spearman correlation coefficient and *p*-value were annotated the left-top corner. **J** KM plot of the TEF gene. The expression group was determined by median value of TEF gene. **K** Violin plot of TEF expression in scRNA-seq dataset between PGNG + / − ECs. The gray line indicated the mean value of TEF expression of PGNG + / − ECs. ****P < 0.0001

We utilized single-cell RNA sequencing (scRNA-seq) analysis to characterize the tumor cells in different gluconeogenesis statuses at single cell resolution. Epithelial cells (ECs) from KIRC tissues were classified into PGNG + / − ECs based on the number of expressed PGNGs genes, which accounted for 16.0% and 84.0% of all ECs, respectively. The CytoTRACE trajectory analysis showed a significant difference between PGNG + / − ECs (Fig. 5A, B), with the predicted order of PGNG + ECs being approximately twofold greater than that of PGNG-ECs (*p* < 0.0001) (Fig. 5C). As CytoTRACE analysis was relied on the number of genes expressed in a cell, this result suggested that the PGNG + ECs were transcriptionally more inactive in the PGNG-ECs. By combining the TCGA KIRC transcriptome results, which showed that PGNGs were widely suppressed in the tumor, we inferred that the PGNG + ECs resembled normal ECs and represented an early stage of tumor initiation.

Next, we analyzed the intercellular communication between PGNG + / − ECs and other cells within the tumor microenvironment. Interestingly, PGNG + ECs exhibited increased activity in both outgoing and incoming interaction strength (Fig. 5D). Furthermore, immune cells showed a stronger interaction in both numbers and strength with PGNG + ECs compared to PGNG-ECs (Fig. 5E), particularly dendritic cells (DCs), macrophages, and T cells. The CXCL12-CXCR4 signaling pathway was found to have a high communication probability from PGNG + ECs to B cells, DCs, macrophages, monocytes, NK cells, and T cells, which was not observed in PGNG-ECs (Fig. 5F). Notably, CXCL12 expression was positively correlated with the PGNG score (*r* = 0.17, *p* = 9.4e-5) in the TCGA KIRC transcriptome dataset (Fig. 5G). Moreover, high CXCL12 expression levels were associated with a favorable prognosis (Fig. 5H), which was consistent with the PGNG score. Similar to CXCL12, TEF expression was also positively correlated with PGNG score, and higher TEF expression indicated a better survival outcome (Fig. 5I, J). In the KIRC scRNA-seq dataset, the expression of TEF in PGNG + ECs was approximately 1.6 times higher than PGNG-ECs (mean values, 0.0478 vs 0.0299, *p* < 0.0001) (Fig. 5K).

## Discussion

Although gluconeogenesis is less explored than glycolysis or TCA cycle by oncologists, it is believed to play an equally crucial role in regulating aerobic glycolysis in cancer cells [7, 33]. PCK, FBPase, and G6Pase are three key enzymes which control gluconeogenesis flux and further influence other metabolic processes, including glycolysis, TCA cycle, and pentose phosphate pathway (PPP) [7, 34]. Several studies have demonstrated that gluconeogenesis was involved not only in cell survival and proliferation but also in epithelial-mesenchymal transition (EMT) and cancer stem cell (CSC) phenotype [35–38]. Nevertheless, gluconeogenesis in kidney tumor, which showed higher levels of metabolism reprogram than other cancer types, remains insufficiently studied [5]. In this study, we systematically analyzed the glycolysis and gluconeogenesis mRNA profiles in KIRC and found that gluconeogenesis genes were under-expressed in tumor. Additionally, we found that the suppression of gluconeogenesis was associated with poor survival outcomes. Furthermore, we identified a molecular signature, referred to as PGNG, which contains 11 tightly correlated genes of gluconeogenesis. Notably, four of rate-limiting enzymes coding genes of gluconeogenesis (FBP1, G6PC, PCK1/2) were at key nodes of the PPI network constructed of the PGNGs, indicating their importance in this metabolic reprogramming. In cancer cells, restoring gluconeogenesis by overexpression of PCK1 strongly blocked the proliferation and survival of kidney tumor cells. Our results highlighted the loss of gluconeogenesis as the crucial metabolic signature in the initiation and development of KIRC, which had negative consequences.

Various mechanisms regulate gluconeogenesis in different cancers, such as transcriptional regulation, epigenetic modification, posttranslational modification, and enzyme activity [39–42]. Our investigation revealed that miR-4477b was a novel endogenous inhibitor of gluconeogenesis in KIRC. Silencing miR-4477b mimicked the gluconeogenesis restoration and pro-apoptotic phenotypes of PCK1 overexpression. MiR-4477b was a novel microRNA which was not fully investigated much less in cancer. Our study also found that miR-4477b inhibited gluconeogenesis by restricting TEF and FBP1, while TEF promoted gluconeogenesis by transcriptionally regulating PCK1 and PCK2. TEF was previously reported to retard bladder cancer cell growth by inhibiting G1/S transition and regulating AKT/FOXOs signaling, which are both closely related to glycolysis/gluconeogenesis homeostasis [43–45]. Interestingly, our research did not identify any other miRNAs previously reported to regulate gluconeogenesis under various conditions (miR-158-5p, miR-351, miR-451, etc.) [46–48], suggesting that the suppression of this process by miR-4477b is specific to KIRC.

At the single-cell level, using CytoTRACE for trajectory analysis, we observed a significant difference in the predicted order of PGNG + / − ECs. Considering that PGNGs were found to be downregulated in KIRC tumor tissue and decreased with TNM stages, we inferred that the PGNG signature was more prominent in the early carcinoma cells, suggesting that the loss of gluconeogenesis may be a key metabolic characteristic in cancer initiation and development. Since the CytoTRACE analysis took the number of genes expressed in a cell into account, the different predicted order of PGNG + / − ECs indicated that PGNG + ECs were transcriptionally more inactivate compared to PGNG-ECs. Surprisingly, the interaction between CXCL12-CXCR4 in PGNG-cancer cells and immune cells was significantly diminished compared to PGNG + cancer cells. Additional research is required to elucidate the underlying mechanism of how gluconeogenesis and immune surveillance are connected.

However, there were several limitations in the current study that required further investigation to better understand the role of gluconeogenesis in KIRC. Firstly, our study was conducted in vitro experiments, and further in vivo testing is required. Additionally, interference with the expression of miR-4477b may affect tumor growth by remodeling the tumor microenvironment, which needs to be tested by histological approaches. Secondly, although we observed changes in gene expression and limited metabolite levels related to gluconeogenesis in KIRC, further comprehensive research is needed to confirm these changes at the metabolite level in vivo and in vitro, including key molecules such as glucose, pyruvic acid, and lactic acid. A metabolomics analysis could provide a more in-depth understanding of the metabolic switch mediated by miR-4477b-TEF axis. Finally, the potential of upstream regulation of miR-4477b, including gene mutations, epigenetic modification which are thought to be related to metabolism reprogramming in KIRC [5], should be the future focus of our research.

## Conclusions

Our study suggests that the deficiency of gluconeogenesis is a key metabolic feature of KIRC, and targeting miR-4477b-TEF axis could effectively restore gluconeogenesis and inhibit the proliferation and progression of KIRC cells.

### Supplementary Information

**Additional file 1: Fig. S1.** Reprogram of glycolysis and gluconeogenesis was crucial for KIRC prognosis. (A) Forest plot of PGNG genes. The hazard ratio of PGNG genes was calculated by the univariate Cox hazard analysis. (B) GSEA plot of PGNG genes. (C) Network of PGNG PPI. The node size indicated the degrees of genes. (D) Barplot of PGNG degree in PPI network.

## Data Availability

The public data of KIRC RNA sequencing prolife and clinical data was available on the TCGA Research Network portal (https://portal.gdc.cancer.gov/). The KIRC scRNA-seq data was obtained from the supplementary materials of the published article (https://www.science.org/doi/10.1126/science.aat1699#supplementary-materials).
