# Supplementary figures and images for: Restoring gluconeogenesis by TEF inhibited proliferation and promoted apoptosis and immune surveillance in kidney renal clear cell carcinoma
Source: Cancer Metab. 2023 Aug 8;11:11. doi: 10.1186/s40170-023-00312-4 (PMC10410999; doi:10.1186/s40170-023-00312-4)

A

HR (95% CI for HR) p.value

|         |                  |         |
|---------|------------------|---------|
| ALDOB   | 0.89 (0.85–0.94) | 3.4e–06 |
| PCK1    | 0.87 (0.83–0.91) | 8.5e–09 |
| ADH6    | 0.87 (0.81–0.94) | 2e–04   |
| G6PC1   | 0.85 (0.81–0.9)  | 5.4e–10 |
| PFKM    | 0.83 (0.72–0.97) | 0.016   |
| FBP1    | 0.81 (0.75–0.88) | 2.6e–07 |
| ALDH1B1 | 0.81 (0.71–0.93) | 0.0032  |
| PCK2    | 0.8 (0.69–0.93)  | 0.0043  |
| DLD     | 0.79 (0.68–0.91) | 0.0014  |
| GALM    | 0.78 (0.65–0.93) | 0.0065  |
| PDHB    | 0.77 (0.63–0.95) | 0.013   |

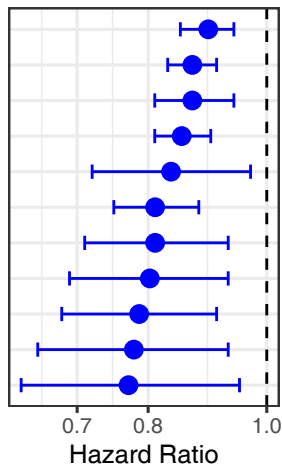

B

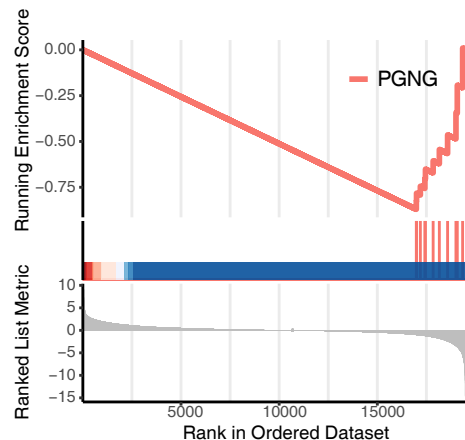

C

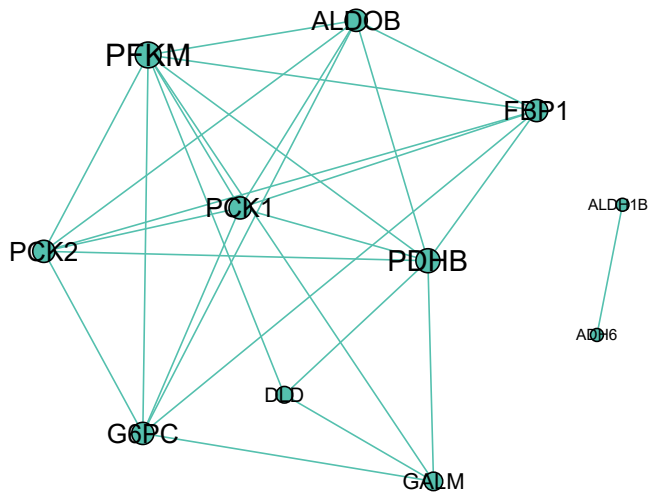

D

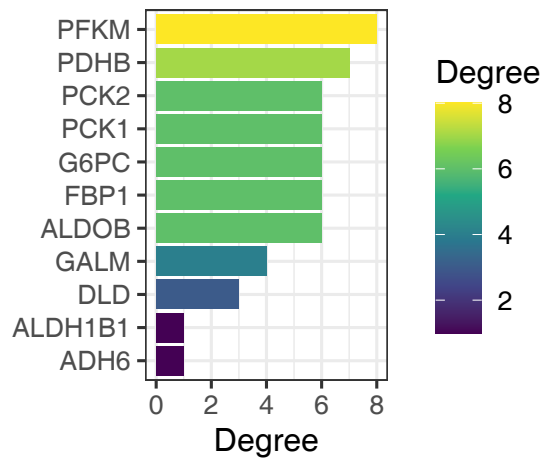

Supplement: Supplementary file 1 — Additional file 1: Fig. S1. Reprogram of glycolysis and gluconeogenesis was crucial for KIRC prognosis. (A) Forest plot of PGNG genes. The hazard ratio of PGNG genes was calculated by the univariate Cox hazard analysis. (B) GSEA plot of PGNG genes. (C) Network of PGNG PPI. The node size indicated the degrees of genes. (D) Barplot of PGNG degree in PPI network. [file 40170_2023_312_MOESM1_ESM.pdf]
